# Impact of Parenteral Lipid Emulsion Components on Cholestatic Liver Disease in Neonates

**DOI:** 10.3390/nu13020508

**Published:** 2021-02-04

**Authors:** Gregory Guthrie, Douglas Burrin

**Affiliations:** USDA/ARS Children’s Nutrition Research Center, Section of Pediatric Gastroenterology, Hepatology and Nutrition, Department of Pediatrics, Baylor College of Medicine, Houston, TX 77030, USA; doug.burrin@usda.gov

**Keywords:** soybean oil, fish oil, medium chain triglycerides, olive oil, parenteral nutrition associated liver disease, preterm infants, vitamin E, lipid emulsions

## Abstract

Total parenteral nutrition (TPN) is a life-saving intervention for infants that are unable to feed by mouth. Infants that remain on TPN for extended periods of time are at risk for the development of liver injury in the form of parenteral nutrition associated cholestasis (PNAC). Current research suggests the lipid component of TPN is a factor in the development of PNAC. Most notably, the fatty acid composition, vitamin E concentration, and presence of phytosterols are believed key mediators of lipid emulsion driven PNAC development. New emulsions comprised of fish oil and medium chain triglycerides show promise for reducing the incidence of PNAC in infants. In this review we will cover the current clinical studies on the benefit of fish oil and medium chain triglyceride containing lipid emulsions on the development of PNAC, the current constituents of lipid emulsions that may modulate the prevalence of PNAC, and potential new supplements to TPN to further reduce the incidence of PNAC.

## 1. Introduction

Total parenteral nutrition (TPN) was first established as a life-saving approach for nutritional support in infants in 1968 [1,2]. Originally, the solution contained only dextrose, amino acids, minerals, and vitamins, as there were no suitable lipid solutions. To overcome this limitation, plasma was given to provide essential fatty acids. However, high carbohydrate administration during TPN can increase hepatic de novo lipogenesis [3] and may predispose individuals to hyperglycemia [4], so the initial formulations were not ideal. Lipid emulsions were created to supply the necessary essential fatty acids to prevent essential fatty acid deficiency (EFAD) and to the meet energy and growth needs [5]. While the provision of TPN containing lipid emulsions has successfully reduced the overall mortality of premature infants and infants that are intractable to enteral feeding, there have been a number of diseases that have arisen from its use. Despite line infections and sepsis being the most common issues with TPN, more complex diseases can arise during TPN use. Cholestatic liver disease historically has been among the common TPN related morbidities in infants.

The general term parenteral nutrition associated liver disease (PNALD) has been used to describe the characteristic set of hepatic and serum abnormalities associated with liver injury attributed to PN administration. The primary marker of PNALD is an elevated serum conjugated bilirubin >2 mg/dL, yet serum alkaline phosphatase (ALP), gamma glutamyl transferase (GGT), alanine amino transferase (ALT) and bile acids are also elevated in infants. Hepatic steatosis and fibrosis are observed histologically in these infants and prolonged PNALD can lead to liver cirrhosis and require liver transplantation. Initial studies into TPN related liver disease in infancy noticed that cholestasis was a common underlying factor, which was observed even prior to the initiation of lipid emulsion supplementation. As cholestasis is a standard hallmark of disease onset, the term parenteral nutrition associated cholestasis (PNAC) was adopted to describe the disease. In recent years, there has been further specification given to the disease. Most infants develop PNAC following intestinal failure, which requires prolonged TPN administration. Given this, the term intestinal failure associated liver disease (IFALD) is more readily used in the literature when the liver disease occurs in infants with intestinal resection. However, many early studies examining the development of PNAC related to lipid emulsions have not differentiated between the causes of TPN treatment. For this reason, we will use the more general term, PNAC, in this review.

The development of PNAC in infants is multifactorial. Given the lack of enteral feeds, gut atrophy can lead to bacterial overgrowth, increased permeability, and bacterial translocation, which can lead to sepsis. This risk of sepsis can be compounded in cases of IFALD, where intestinal resection leads to inflammation and greater potential for increased intestinal permeability. Sepsis can also occur through central line infections and lead to PNAC. There is also strong relationship with infant prematurity and associated immature liver function leading to PNAC. However, in many cases these factors are difficult or impossible to modify and avoid. Thus, lipid emulsion composition is a modifiable factor related to PNAC that can reduce the likelihood of disease onset and perhaps disease resolution.

This review reports on the current knowledge of lipid emulsions and how they impact the development of cholestatic liver disease in infants. The potential mechanisms that drive cholestatic liver disease are discussed, as well as, potential ingredients that may be added to lipid emulsions to reduce the development of cholestatic liver disease.

## 2. Lipid Emulsion Composition and Clinical Effects

In enteral feeding, dietary lipid is packaged in chylomicrons in the intestine. Lipid emulsions mimic chylomicron composition which contains a hydrophilic outer domain and a hydrophobic inner domain. The standard components found in all lipid emulsions are glycerol (2.25–2.5 g/100 mL), egg phospholipid (1.2 g/100 mL), to function as an emulsifier, and a triglyceride rich core. The first successful lipid emulsions used soy oil to supply the triglycerides. There have been more advanced lipid emulsions created in the past 20 years that offer additional lipid sources with varied fatty acid composition which are believed to provide a higher concentration of metabolically beneficial fatty acids. The two most relevant new lipid emulsions in use for infants are fish oil and multi-component oils comprised of soy oil, fish oil, olive oil, and coconut oil.

### 2.1. Clinical Outcomes with Soy Oil-Based Lipid Emulsions

Soy-based lipid emulsions (SO-LE) have been the principal source of lipids for TPN in the US since they were initially made available in 1972. SO-LE is primarily comprised of omega-6 fatty acids with linoleic acid constituting approximately 50% and the omega-3 fatty acid, alpha linoleic acid (ALA) only making up 9%. There are minimal amounts of long chain fatty acids arachidonic acid (ARA), eicosapentanoic acid (EPA), and docosahexaenoic acid (DHA). Overall, the ratio of omega-6 to omega-3 fatty acids is approximately 7:1. In addition to fatty acids, SO-LE has other biologically active compounds, namely phytosterols, which are cholesterol-like molecules that are ubiquitous to plant-based lipids [6]. SO-LE also contains some vitamin E in the form of γ-tocopherol, which is less bioavailable than α-tocopherol, the predominant biologically active vitamin E form in the body [7].

The short-term use of SO-LE in TPN is safe and effective in improving the survival of infants that otherwise cannot be fed enterally. The development of PNAC was observed in multiple studies by the late 1970′s; however, there was no direct connection between PNAC and SO-LE [8,9,10,11,12]. Allardyce et al. made the initial observation that PNAC occurred in 10 of 18 patients receiving 3 g/kg/d SO-LE compared to only 1 of 17 patients receiving a reduced lipid load of 1 g/kg/d [13]. Subsequent studies support that reduced SO-LE lipid load can decrease or slow the progression of PNAC in infants [14,15,16,17]; however, there is an increased risk for development of EFAD, which makes this approach unfeasible for long-term TPN support [14]. PNAC gradually occurs in infants receiving SO-LE. The incidence rate of PNAC from SO-LE varies remarkably between centers from 7–85% and is likely due to the underlying cause requiring TPN administration and the duration it is administered [8,10,18,19,20]. A large, multi-center study of 6543 infants receiving TPN containing SO-LE found that infants receiving TPN for 14–28 days had a 14% incidence of PNAC [20]. The percentage of infants with PNAC increased as a factor of time with 85% of infants still receiving TPN at >100 days developing the disease.

There is no consensus on the specific component of SO-LE that leads to PNAC development. Multiple studies researching the function of omega-6 fatty acids suggest that high omega-6 fatty acids have a negative impact on inflammation and oxidative stress, which make them possible causes for PNAC [21,22,23]. In addition, clinical studies have shown a strong correlation between the phytosterols present in SO-LE and PNAC, which is supported by cell culture and animal model studies [24,25,26,27,28]. There is also research to support that low bioavailable vitamin E concentrations of SO-LE is insufficient to protect from oxidative stress during TPN administration [29,30]. It is likely that the development of PNAC from SO-LE is multifactorial and may involve the interplay of all these suggested mediators.

### 2.2. Clinical Outcomes with Fish Oil-Based Lipid Emulsions

Fish oil lipid emulsion (FO-LE) is pure fish oil that contains high concentrations of EPA (19%), DHA (12%) and low concentrations of LA (4.4%), ALA (1.8%), and ARA (1–4%). Unlike SO-LE, FO-LE is supplemented with vitamin E in the form of α-tocopherol at approximately 200 mg/L and since there are no plant oil sources in FO-LE, it has a minimally detectable concentration of phytosterols. The first use of FO-LE as a sole source of lipid was in the prevention of essential fatty acid deficiency in a patient with soy-allergy receiving parenteral nutrition in 2005 [31]. The first study to directly address the role of FO-LE in resolving PNALD was published a year later in 2006 [32]. Two infants that had developed PNALD following short-bowel resection were administered FO-LE at a dosage of 1 g/kg/d. The cholestasis resolved at 60 days of treatment. Most case studies and single-center studies support a high resolution rate of PNAC from infants switched to FO-LE [33,34,35,36,37,38,39], with one study finding a positive resolution of greater than 80% of patients [35]. One limitation to these studies was that SO-LE was administered at 3.0 g/kg/d and FO-LE was administered at 1.0 g/kg/d. One double-blind, placebo controlled study directly compared the resolution of PNAC in infants receiving either SO-LE vs. FO-LE at 1.5 g/kg/d. Infants that had developed PNAC were randomized to either TPN containing FO-LE or SO-LE for a minimum of 2 weeks [40]. FO-LE did not resolve PNAC, but did stop further progression of the disease as assessed by direct bilirubin levels; however, once infants were transitioned to enteral feeds, those receiving FO-LE had a more rapid decrease in direct bilirubin than those transitioned to enteral feeds that were receiving SO-LE. Overall, this result suggests there is still a net positive effect from receiving FO-LE over SO-LE. Nehra et al. addressed whether FO-LE started at the beginning of TPN administration, rather than onset of PNAC, would perform better than SO-LE with matched lipid dose [41]. At a lipid dosage of 1 g/kg/d, this study failed to see any difference in PNAC prevalence between lipid emulsions, as neither treatment groups developed PNAC. As such, there are no studies to support the use of FO-LE to prevent the onset of PNAC, but the majority of research does support efficacy at resolving PNAC once established. This clinical evidence forms the basis on which the Federal Drug Administration (FDA) has approved Omegaven for the treatment of PNAC in infants.

There were initial concerns with the use of low-dose FO-LE on the development of essential fatty acid deficiency given the very high EPA and DHA and very low LA and ALA fatty acid composition. However, given at the appropriate daily dose (1 g/kg) FO-LE is effective at maintaining essential fatty acid concentrations [42], and in one case study, shown effective at reversing EFAD in a malnourished infant at a dose of 1.5 g/kg/d [43]. It has been suggested the provision of enough DHA and ARA is sufficient to prevent EFAD through retroconversion pathways to form other shorter chain fatty acids [44]. There have been concerns raised over abnormal platelet function from ARA deficiency during FO-LE administration in infants and piglets, so there still may be issues with the adequacy of FO-LE [45,46]. However, infants do not appear to have any issues with growth restriction when administered 1.0 g/kg/d FO-LE [47,48,49]. There are still questions about the long-term viability of using FO-LE and its impact on very preterm infants. Sufficient large-scale clinical trials are needed to address the current gaps in knowledge for FO-LE as a sole-source lipid for infants.

### 2.3. Clinical Outcomes with Mixed Oil-Based Lipid Emulsions

The term “mixed oil lipid emulsions” is used in the literature as a very general term for any emulsion that is not comprised of a single-source lipid. This designation has been a confounding factor as the lipid source mixtures can be highly variable. Some mixed oil emulsions are comprised of two plant source lipids, such as soy oil and olive oil. Other lipid emulsions can be made of soy oil and fish oil. In addition, there can be multi-component lipid emulsions comprised of three or four lipid sources. For the purpose of this review, the mixed lipid oil emulsion that will be in focus is four-source multicomponent lipid emulsions (MO-LE). MO-LE is comprised of medium chain triglycerides (30%), soy oil (30%), olive oil (25%), and fish oil (15%). SMOFlipid (Fresenius Kabi) is the predominant MO-LE in use in Europe and has been approved for use in adults in the US and has off-label approval for use in infants.

MO-LE fatty acid profile is an intermediate between the high LA of SO-LE and high DHA and EPA of FO-LE. MO-LE contains 18% LA, 2.5% ALA, 3% EPA, 2% DHA, 0.15–0.6% ARA, and the medium chain triglycerides caprylic acid (16%), caproic acid (11%) [50]. This composition gives MO-LE an omega-6 to omega-3 ratio of 2.5:1, much lower than that of SO-LE. Even though MO-LE contains one-third the concentration of soy oil as SO-LE, it still contains about half the total phytosterol concentration, given olive oil supplies additional phytosterol [51]. Similar to FO-LE, MO-LE is supplemented with approximately 200 mg/L α-tocopherol. Unlike FO-LE, MO-FE can be administered at concentrations up to 3.5 g/kg/d in infants.

The results of clinical trials on the efficacy of MO-FE over SO-LE in resolving PNAC have been mixed. Some small size clinical studies show a benefit from MO-LE [50,52,53,54,55], as well as some larger randomized control trial (RCT) studies [56]. However, when data is grouped for meta-analyses that have stringent exclusion criteria MO-FE has failed to show a positive effect on treatment for PNAC [57,58]. Other meta-analysis that include less stringent inclusion criteria have found a significant positive effect with MO-FE [59,60]. In the most recent Cochrane meta-analysis, eleven studies were analyzed comparing MO-FE vs. SO-LE in infants without surgical or preexisting conditions on the efficacy of cholestasis prevention [58]. Of these studies, five observed no effect in the difference between SO-LE and MO-LE on conjugated bilirubin concentrations [51,61,62,63,64]. A second comparison was performed between MO-LE and SO-LE in infants with PNAC and preexisting surgical procedures. Based on the criteria, only a single multicenter RCT study was included [56]. Over an eight-week period, infants with PNAC receiving MO-LE had a reduction in conjugated bilirubin from 35 to 22 µmol/L. Infants that remained on SO-LE had an increase of conjugated bilirubin from 36 to 69 µmol/L. For a more detailed breakdown of each study, Kapoor et al. systematic meta-analyses provide a thorough examination of the main outcomes comparing SO-LE and MO-LE [58,65].

More recent studies have promising results with MO-LE. Lam et al. found that long-term exposure (>4 weeks) to MO-LE resulted in lower conjugated bilirubin than SO-LE [66]. Torgalkar et al. did not see a significant reduction in cholestasis, but did see a lower odds ratio with MO-LE use [67]; however, the authors stated this may have been due to the reduced time of treatment in the MO-LE group. Interestingly, a recent study by Ferguson el al. looked at 107 patients in a retrospective cohort and found that 44.8% with intestinal failure developed PNAC while receiving SO-LE and only 30% of patients receiving MO-LE [68]. In their multivariate analysis they found that after adjustment for cofounding factors, the improved performance of MO-LE could not be attributed to the lipid emulsion. Similar to FO-LE studies, larger, multi-center RCT trials are needed to get a better impression of the overall benefit of MO-LE compared to SO-LE. Given that MO-LE is now readily available off-label, its use for TPN will become more ubiquitous and allow for high quality reports on its overall efficacy.

### 2.4. Preclinical Studies with Lipid Emulsions

Animal models of parenteral nutrition strongly support the clinical findings for the role lipid emulsions play in the development of PNAC. An important distinction between human and animal studies is that, thus far, animal studies have not looked at the ability of FO-LE and MO-LE to resolve PNAC, but rather whether they can prevent the development of PNAC. As discussed above, this has been a considerable limitation in clinical studies, given the relatively low overall incidence rate of PNAC in infants in studies trying to test the efficacy of new generation lipid emulsions compared to SO-LE.

In adult mouse models, mice that are enterally fed PN solution containing amino acids and glucose while being administered SO-LE intravenously rapidly develop hepatic steatosis [69,70,71,72,73]. The administration of FO-LE does not lead to steatosis in this model [71,73]. There has been some mixed data on the effect of MO-LE, with more recent mouse studies suggesting that the addition of MCT oil to lipid emulsions can reduce the onset of steatosis and inflammation [30]. However, cholestasis does not occur in this model, likely due to the inclusion of some enteral feeds. In mouse studies that have used TPN instead of split enteral/parenteral feeding, SO-LE alone does not cause hepatocellular injury, nor cholestasis when given TPN for up to 28 days. When mice are given a co-treatment of dextran sodium sulfate (DSS), to cause intestinal inflammation prior to administration of TPN for 28 days, they do develop cholestasis and display increased markers of liver injury [27,28,74]. In agreement with clinical studies, mice that are administered FO-LE following DSS administration do not develop cholestasis or have an increase in markers of liver injury [27,28]. These studies strongly suggest that there is an inflammatory-mediated event through activation of IL-1β during TPN that drives the disease development [28].

One limitation to the use of mice in TPN studies is, given their size, the studies need to be performed after they have reached maturity. Given this, the piglet has been a very useful model for studying neonatal PNAC development because of their large birth size and their metabolic needs are closer to that of humans. Piglets develop cholestasis from TPN within 2 weeks of SO-LE administration similar to human infants receiving TPN [51,75,76,77,78,79,80,81,82,83,84]. In piglets, this does not require the addition of any type of inflammatory insult, such as DSS. The cholestasis spontaneously occurs with a rapid increase of direct bilirubin at day 10 of TPN administration [75]. Administration of FO-LE and MO-LE effectively prevent the development of cholestasis in piglets [51,84,85]. Unlike mouse studies, there does not appear to be a benefit of FO-LE over MO-LE in preventing the development of steatosis. Both emulsions are equally superior to SO-LE.

## 3. Lipid Emulsion Components That Modify PNAC Incidence

### 3.1. Phytosterols

Phytosterols are a collective term for a group a sterols that are structurally similar to mammalian cholesterol. The predominant phytosterols present in SO-LE and MO-LE are β-sitosterol, campesterol, and stigmasterol. Under normal feeding, limited amounts of phytosterols consumed enterally are absorbed or accumulate in the systemic circulation due to the action of transporters, heterodimeric partners ATP-binding cassette transporters-G5 and -G8, expressed in the liver and intestinal epithelium; these transporters function to export phytosterols into the biliary system and gut lumen where they are excreted in feces [86,87]. During TPN, phytosterols enter the systemic circulation and bypass intestinal uptake and export into the gut lumen, yet phytosterols are taken up and accumulate in the liver because they are not excreted into the bile due to cessation of biliary flow. As a result, phytosterols in the blood can accumulate to levels exceeding 1 mM during long-term TPN administration, as was shown by Clayton et al. who first observed a positive correlation between the severity of PNAC, as determined by direct bilirubin concentrations, and the concentration of plasma phytosterols [24].

There are currently two proposed mechanisms for how phytosterols may cause PNAC. Carter et al. showed that the phytosterol, stigmasterol, can directly inhibit ligand binding to the nuclear hormone receptor, farnesoid x receptor (FXR) in cell culture [25], and others have shown similar FXR target gene repression in primary hepatocytes [51]. FXR is the key regulator of bile acid transport and synthesis. In the liver, FXR upregulates the bile salt export pump (BSEP), which facilitates the hepatobiliary transport of bile acids into the bile ducts. In the intestine, FXR upregulates the enterokine fibroblast growth factor-19 (FGF-19), which circulates via portal blood to the liver where is binds to its cognate heterodimeric receptors FGF receptor 4 (FGFR4) and β-klotho. The subsequent activation of the ERK-signaling pathway leads to repression of the cytochrome P450 7a1 (Cyp7a1). Cyp7a1 is the rate-limiting enzyme on bile acid synthesis. Through suppression of Cyp7a1 transcription by FGF19, FXR can reduce bile acid synthesis in the liver. Studies in TPN-fed neonatal piglets showed that the suppression of biliary flow leads to reduced circulating FGF19 and that enteral administration of the bile acid, chenodeoxycholic acid, a potent FXR agonist, increased plasma FGF19 and prevented cholestasis. This finding supports a role for the disruption of the FXR-FGF19 signaling axis in the onset of PNAC [76].

The second proposed mechanism is that phytosterols can enhance the inflammatory response in macrophages [26,27,28]. Specifically, they enhance the expression of the cytokine IL-1β. Interestingly, phytosterols cannot upregulate IL-1β without an initial exposure to endotoxin. In healthy mice, chemical injury to the intestine by treatment with dextran sulfate sodium prior to administration of TPN containing phytosterols promotes PNAC; yet knock out mice with deletion of the toll like receptor 4 (TLR4) gene, the primary endotoxin receptor on Kupffer cells, do not develop PNAC following TPN administration [27]. This model fits well with the observation that the incidence rates of PNAC are greatest in infants that have had intestinal failure, as there can be increased leakage of endotoxin from the gut to the liver [88]. When neonatal piglets [29], or adult mice [30], are administered FO-LE supplemented with phytosterols, neither species shows any markers of PNAC development. This may be due to the anti-inflammatory properties of omega-3 fatty acids to suppress endotoxin receptors and prevent phytosterols from enhancing those inflammatory pathways [89].

### 3.2. Vitamin E

Vitamin E is a general term for a set of molecules termed tocopherols and tocotrienols that exist naturally as four differing isoforms, -α, -β, -γ, -δ. The main biological function of vitamin E is to prevent lipid peroxidation of cellular membranes [90]. The isoforms all confer the same antioxidant activity yet differ significantly in overall biological activity with α-tocopherol being the most active [91]. This is due to the affinity α-tocopherol has for the tocopherol transport protein (TTP). TTP protects α-tocopherol from rapid degradation and clearance in the liver. It is of note that plant-based oils such as SO-LE predominantly contain the γ-tocopherol isoform. As such, SO-LE provides very limited antioxidant protection of lipids. SO-LE is not supplemented with vitamin E as most of the fatty acids are 18-carbon in length with a low level of unsaturation (2 double bonds). FO-LE and MO-LE also have low naturally occurring concentrations of vitamin E; however, FO-LE and MO-LE consist of highly unsaturated fatty acids that make them susceptible to oxidation and breakdown into volatile compounds [92]. Due to this, they have been supplemented with natural α-tocopherol to protect from peroxidation of the very long chain fatty acids present in fish oil.

Preterm infants have an increased susceptibility to oxidant stress due to immature antioxidant mechanisms [93,94]. This puts them at particular risk as cellular oxidative stress is an important factor in the development of liver disease in conjunction with hepatic steatosis [95,96]. In the case of PNAC, the source of oxidants can be either external, from the lipid emulsion [97,98], or internal from overload of normal lipid metabolism via mitochondrial β-oxidation to peroxisomal β-oxidation and microsomal ω-oxidation [99,100,101]. Cholestasis can also promote oxidative stress through bile salts either activating cellular apoptotic pathways [102] or inhibiting mitochondrial β-oxidation [103] causing a shift to the other oxidation pathways mentioned above.

The use of vitamin E to resolve nonalcoholic liver disease has had mixed results. In adults, supplemental vitamin E can reduce hepatic inflammation, steatosis, and cellular ballooning [104]; however, in children a similar study was performed giving children oral doses of supplemental vitamin E. The trial, called the TONIC (Treatment for Non-Alcoholic Fatty Liver Disease (NAFLD) in Children) trial, failed to see any improvement compared to placebo [105]. There have been no clinical studies to test the efficacy of added supplemental vitamin E to SO-LE in the prevention of PNAC in infants. The efficacy of supplemental vitamin E to prevent PNAC has been studied in TPN-fed neonatal piglets, but with mixed results. In premature, neonatal piglets administered TPN containing SO-LE at 5 g/kg/d, α-tocopherol supplemented to concentrations present in FO-LE prevented an increase in direct bilirubin and bile acid concentrations, similar to the effect of FO-LE in the study [29]. In two term neonatal piglet TPN studies where SO-LE was given at a higher lipid load (10 g/kg/d), supplemental vitamin E, as α-tocopherol, failed to prevent PNAC based on serum conjugated bilirubin and serum bile acid levels [83,106]. An interesting observation made in both studies, was that the vitamin E supplemented groups showed no change in serum markers of oxidant stress relative to the standard SO-LE treatment groups. Neither study examined hepatic or red blood cell oxidant status, so it is not clear in either study whether supplemental vitamin E had any modifying effect to oxidant stress. In the mouse model, the use of supplemental vitamin E has had more consistent positive effects. Fell et at. administered vitamin E supplemented SO-LE enterally to adult mice which prevented steatosis and maintained healthy hepatic architecture compared to regular SO-LE [70]. In a similar study, enteral administration of FO-LE was supplemented with vitamin E for 19 days [30]. The mice were then treated with lipopolysaccharide (LPS) to induce an inflammatory response. The FO-LE vitamin E supplemented mice did not develop hepatic steatosis during the study and had a reduced cytokine response to LPS challenge for Il-6 and Tnf-α. A caveat to these studies is that the mice were adults during treatment and based on the clinical vitamin E trials, there may be an age-dependent benefit to vitamin E supplementation. The role of vitamin E in preventing PNAC still remains unclear given these inconsistent results in animal models.

### 3.3. Fatty Acid Composition

The fatty acid composition of SO-LE, FO-LE, and MO-LE differ in the ratios for omega-6 to omega-3 fatty acids, the chain length of fatty acids and the degree of chain saturation between the fatty acids. Each fatty acid characteristic imparts relevant biological effects that can either promote or prevent an environment that will cause cholestasis and liver injury.

#### 3.3.1. Omega-6 and Omega-3 Fatty Acids

The 18-carbon fatty acids linoleic acid (LA, omega-6) and linolenic acid (ALA, omega-3) present in intravenous lipid emulsions prevent the development of essential fatty acid deficiency. However, a high ratio of omega-6 to omega-3 fatty acids positively correlates with elevated serum inflammatory markers [21]. The underlying reason for this observation has been ascribed to the relative pro-inflammatory effects of omega-6 fatty acids and the anti-inflammatory effects of omega-3 fatty acids (citations). LA and ALA undergo chain elongation to the omega-6 fatty acid, arachidonic acid (ARA) and omega-3 fatty acids, EPA and DHA, respectively. These fatty acids are important substrates for the production of bioactive molecules including prostaglandins and leukotrienes for ARA and EPA and the resolvins and protectins, for EPA and DHA.

ARA, EPA, and DHA are incorporated into phospholipids. In response to an environmental stimuli, they are released from the membrane via phospholipases and are substrates for the enzymes cyclooxygenases (prostaglandin synthesis) or lipoxygenases (leukotriene synthesis). ARA is converted to several different prostaglandins, but one of the more predominant variants is PGE_2_. PGE_2_ can stimulate inflammation though activating signaling cascades in immune cells. In mast cells, PGE_2_ activates the release of histamines and interleukin-6 [107,108]. In helper T cells, PGE_2_ facilitates differentiation into proinflammatory Th1 cells [109] and expansion of Th17 cells [109,110]. Another prostaglandin metabolite of ARA is thromboxane A2 (TXA_2_). In the liver, TXA_2_ regulated release of tumor necrosis factor alpha (TNF-α) from resident macrophages (Kupffer cells) leads to microcirculatory dysfunction [111]. The lipoxygenase synthesized leukotriene B4 (LTB_4_) is also derived from AA. LTB_4_ is a leukocyte modulator of inflammatory response. In neutrophils, LTB_4_ activates chemotaxis and proinflammatory chemokine and cytokine production [112]. Omega-3 fatty acid EPA forms similar prostaglandin and leukotriene molecules, such as PGE_3_ and TXA_3_ and LTB_5_. EPA and ARA compete for access to the same enzymes in prostaglandin and leukotriene biosynthesis. A greater incorporation of EPA, will reduce the formation of ARA-derived prostaglandins and leukotrienes [113,114,115]. Also, PGE_3_ has much lower affinity for some surface receptors leading to a weaker cellular response when there is a greater concentration of EPA-derived prostaglandins [116]. Unique to omega-3 fatty acids is the formation of resolvins and protectins. DHA forms d-series resolvins and EPA forms e-series resolvins. Resolvins have multiple anti-inflammatory properties. EPA derived resolvins downregulate leukocyte adhesion, ADP-dependent platelet activation [117], and stimulates IL-10 production [118]. DHA derived resolvins enhance bacterial scavenging and clearance [119], protect against proinflammatory glutathione conjugates during oxidative stress [120], and protect from polymorphonuclear leukocyte-mediated organ injury [121]. Protectins can promote T cell apoptosis and resolution [122]. Independent of the bioactive metabolites they form fatty acids may also directly initiate inflammatory processes. Palmitate, a saturated fatty acid, can bind to toll like receptor 4 (TLR4). DHA can inhibit palmitate TLR4 binding and suppress the inflammatory response [89].

In addition to an anti-inflammatory effect, EPA and DHA have a positive effect on lipid utilization and glucose metabolism. Dietary supplementation of EPA and DHA can activate the transcription factor peroxisome proliferator activated receptor alpha (PPARα) [123]. PPARα upregulates carnitine palmitoyltransferase 1A, which facilitates mitochondrial transport of fatty acids and pyruvate hydrogenase kinase 4, which facilitates glucose oxidation [124]. PPARα can also decrease hepatic fibrosis caused by hepatic steatosis [125]. In Kupffer cells, omega-3 fatty acids can increase the action of G-protein coupled receptor 120 (GPR120). GPR120 suppresses the lipogenic nuclear hormone receptor PPARγ [71].

#### 3.3.2. Medium Chain Triglycerides

Medium chain triglycerides (MCT) were first incorporated into lipid emulsions in the 1980s as way to reduce the omega-6 fatty acid content in SO-LEs [126,127]. MCTs are rapidly metabolized in the liver as they do not require carnitine for transport into the mitochondria; as such they do not accumulate in the liver [128]. Given their rapid disposal as an energy source, they also function to spare the metabolism of other fatty acids, such as DHA and EPA and therefore support incorporation of these fatty acids in phospholipids. MCTs display a protective role in animal models of alcoholic liver injury [129] and NALFD [130]. MCTs have anti-inflammatory properties [69,131,132] which may function through suppression of TLR4 signaling in the liver [133].

## 4. Potential Components to Reduce PNAC Incidence

### 4.1. Choline

The primary metabolic function of choline is to supply carbon in the form of betaine for one carbon metabolism [134]. However, choline is also an important source for phospholipids, which play an important role in membrane integrity and function. The liver is a major site of choline metabolism, where it is found in phospholipid form as phosphatidylcholine [135]. In adults, dietary restriction of choline leads to the accumulation of hepatic lipids [136]. In animal models, the use of the methionine-choline-deficient diet (MCD), results in hepatic lipid accumulation, inflammation, and fibrosis, characteristic hallmarks of non-alcoholic fatty liver disease (NAFLD) [137]. Choline deficiency may directly lead to NALFD development through altered hepatic lipid clearance and hepatic de novo lipogenesis. Choline deficiency may suppresses very low density lipoprotein (VLDL) secretion [138], HDL-mediated reverse cholesterol transport through reduced triglyceride synthesis [139], increase lipid droplet size through increased perilipin2 association in lipid droplets [140], and altered ER structure which leads to elevated sterol regulatory element binding protein (SREBP-1) to directly increase cellular lipid accumulation [141].

Infants have a recommended enteral daily allowance (RDA) of 125 mg choline from 0–6 months of age. However, there is no established allowance for infants during parenteral feeding [142] and importantly choline is not supplemented in current parenteral nutrition solutions. SO-LE contains approximately 24 ± 6 nmol/mL free choline and 11,630 ± 552 nmol/mL phospholipid bound choline, which is primarily derived from the egg phospholipid emulsifier in the emulsion [143]. These concentrations fall below the amount necessary to reach enteral RDAs. Due to this, low serum choline concentrations have been observed in both adults [143,144,145] and children [146,147,148,149] receiving long-term parenteral nutrition. In adults receiving PN that have developed PNALD, adding supplemental choline over 24 weeks can reduce markers of hepatic injury including ALT, AST, and ALP [144]. There are no clinical studies on the role of supplemental choline in infants to prevent PNALD. Infants have higher free choline levels compared to adults, which may reflect a higher metabolic requirement for choline during infancy [148]. There are also limited neonatal animal models for the role choline in TPN. Zhu et al. treated 4 week old rat pups with TPN containing 6.67 ± 0.30 mg/d of choline (normal choline) or TPN supplemented with choline containing 59.05 ± 2.28 mg/d [150]. At the end of administration of TPN for 7 days, there was an increase in markers of PNAC including direct bilirubin, ALT, and serum bile acids in the normal choline group. The supplemented choline group had lower values for all PNAC markers; however, the values for direct bilirubin and serum bile acids were slightly elevated compared to an enteral control group. The same research group also found the choline supplemented to TPN can improve intestinal motility [151]. This may be of particular importance given the gut atrophy that occurs during TPN administration. There is considerable interest in the potential benefit of supplemental choline for TPN, as the American Society of Parenteral and Enteral Nutrition published recommendations for parenteral nutrition products recommending that choline be made available in an I.V. form to facilitate administration during TPN [142].

### 4.2. Carnitine

Carnitine is a non-essential amino acid derived from lysine and methionine in the liver and kidney. Carnitine is primarily involved in the transport of long-chain fatty acids across the mitochondrial membrane for β-oxidation through the formation of acylcarnitines. Carnitine also has a secondary function to scavenge intermediates of amino acids and facilitate their clearance [152], as well as necessary for the biosynthesis of DHA [153]. Infants have a rapid increase in carnitine stores in the third trimester and infants born prematurely have significantly less muscle storage of carnitine than term born infants [154]. Unlike adults, the capacity to synthesize carnitine appears to be limited in infants. Human and cow’s milk both contain carnitine and enterally fed infants maintain adequate carnitine stores. There is no supplementation of carnitine in PN solutions. As such, premature infants receiving PN are carnitine deficient [155]. This effect is not species specific, as carnitine deficiency occurs in preterm piglets receiving PN as well [156]. This reduction in carnitine status is also seen in the liver and muscle of piglets. In the liver, there are large decreases in the 2-carbon, 3-carbon, and 4-carbon carnitines.

Carnitine deficiency leads to decreased concentrations of ketone byproducts of β-oxidation, which are important for brain and nervous system maturation [157]. Given the impaired utilization of fatty acids, there is accumulation of lipid in the liver and there can be elevated triglycerides [158]. Impaired lipid clearance can make infants more sensitive to lipid overload from PN administration. The failure to utilize lipids as an energy source can subsequently result in reduced weight gain and growth [159]. Carnitine deficiency may also lead to cholestasis. A case report on a Korean premature infant with a carnitine transport defect that presented with cholestatic jaundice was resolved with oral carnitine supplementation [160].

Provision of supplemental carnitine can increase the serum levels of carnitine [161,162,163]. Infants that have carnitine deficiency from genetic disorders have rapid recovery from supplemental carnitine [164]. The benefit of carnitine supplementation for infants on PN is still not clear. There should be some caution with the use of parenteral carnitine. In adult critically ill patients, parenteral carnitine has been shown to either reduce fat oxidation and increase protein oxidation [165] or help maintain muscle glycogen stores, but not improve lipid or energy metabolism [166]. Currently, animal model studies are necessary to determine if TPN supplementation of carnitine is tolerated and beneficial.

### 4.3. N-acetylcysteine

Cysteine is a conditionally essential amino acid in infants. Cysteine is derived either preformed in the diet or synthesized from methionine. For synthesis, the enzyme γ-cystathionase (CTH) is necessary to metabolize the cysteine precursor, cystathionine. CTH expression is very low in the second trimester but increases rapidly after birth in infants. Hepatic concentrations reach maturity at approximately 3 three months of age [167]. Premature infants have markedly reduced CTH and the reduced CTH correlates with elevated levels of serum cystathionine [168] and reduced synthesis of cysteine is observed in preterm infants receiving TPN [169]. Cysteine concentrations play an important role in antioxidant functions within the cell, through its source as a constituent amino acid of glutathione (GSH). The primary function of GSH is in scavenging free radicals to maintain redox homeostasis. The rate-limiting step of GSH synthesis is the formation of γ-glutamylcysteine from L-glutamate and cysteine [170]. The low concentrations of cysteine in preterm infants is associated with low GSH concentrations [168]. Therefore, low concentrations of cysteine can increase hepatic exposure to oxidative stress [171].

GSH synthesized in hepatocytes is transported to both the systemic circulation and into bile through multidrug resistance-associated proteins (MRP)—1, and MRP-2, respectively [172,173,174,175]. A secondary function of GSH specific to the liver is enhancing bile flow by generating a concentration gradient for the efflux of bile [176]. In the cholangiocyte, GSH is a substrate for the enzyme γ-glutamyl transferase which converts GSH to glutamate [177,178,179]. Glutamate is utilized as an energy source in the cholangiocytes. In infants with PNAC, the activation of bile flow and providing and energy source for injured cholangiocytes may be more useful in resolving cholestasis than providing antioxidant protection.

In the recent guidelines on pediatric parenteral nutrition for amino acids by the European Society for Pediatric Gastroenterology, Hepatology, and Nutrition a recommendation for cysteine supplementation at 50–70 mg/kg/d was given for preterm neonates [180]. This recommendation is challenging as cysteine has poor stability in solution. Originally, methionine was supplied in concentrations to maintain the needed methionine for one-carbon metabolism or shunting towards the synthesis of cysteine via the transulfuration pathway. In preterm infants with impaired cysteine synthesis, excess methionine can accumulate, as one-carbon metabolism is tightly regulated in the liver. Elevations of methionine lead to depletion of hepatic ATP [181] which can increase oxidation of GSH. Due to this, in animal models, administering high concentrations of methionine can lead to cholestasis and hepatocellular injury [182].

*N*-acetylcysteine (NAC) is a stable, soluble compound that is safe in infants [183,184,185] that can increase GSH [185]. NAC is effective in reducing oxidant stress in non-TPN related disease such as acetaminophen and non-acetaminophen induced acute liver failure [186,187]. A current phase 2 trial is in progress for the treatment of another type of neonatal obstructive cholestasis, biliary atresia [188]. The trial is testing the efficacy of NAC to restore bile flow in these infants following surgical intervention, which would have direct implications to the ability of NAC to restore bile flow in PNAC as well. There is limited data on the use of NAC in preventing TPN associated diseases. A single case study of three infants receiving TPN is available [185]. Infants were provided with increasing dosages of NAC every month. By 10 months of NAC administration, conjugated bilirubin and serum bile acids were reduced. Unfortunately, there are no clinical or animal model studies giving NAC at the onset of TPN to see if it prevents the development of PNAC. Follow up studies on NACs ability to resolve and studies on NACs capacity to prevent PNAC are needed.

## 5. Conclusions

In the past 20 years, advancements in lipid emulsions for TPN have made great strides to reduce the incidence of PNAC. FO-LE and MO-LE show promise for replacing SO-LE as primary sources of lipid for TPN in infants. What is clear though is that there is still more we need to learn about the molecular effects the lipid components have on protecting and promoting injury to the liver. The role of phytosterols as drivers of cholestatic liver disease is likely, but studies on the role of omega-6 fatty acid lipid emulsions devoid of phytosterols are needed to separate the role of one factor from the other. Vitamin E supplementation as α-tocopherol may only have limited benefit in infants and animal studies need to better address the disparity between adult and infant response to vitamin E supplementation. There is a limited understanding currently of how different component parenteral fatty acids and their mixtures modify inflammatory and metabolic responses. Furthermore, research is needed to examine other supplemental ingredients that can be added to lipid emulsions to promote improved outcomes.
